# Chilean *Prosopis* Mesocarp Flour: Phenolic Profiling and Antioxidant Activity

**DOI:** 10.3390/molecules20047017

**Published:** 2015-04-17

**Authors:** Guillermo Schmeda-Hirschmann, Cristina Quispe, Maria del Pilar C. Soriano, Cristina Theoduloz, Felipe Jiménez-Aspée, Maria Jorgelina Pérez, Ana Soledad Cuello, Maria Inés Isla

**Affiliations:** 1 Laboratorio de Química de Productos Naturales, Instituto de Química de Recursos Naturales, Universidad de Talca, Casilla 747, Talca 3460000, Chile; E-Mails: mcaramantin@utalca.cl (M.P.C.S.); fjimenez@utalca.cl (F.J.-A.); 2 Facultad de Ciencias de la Salud, Universidad Arturo Prat, Casilla 121, Iquique 1110939, Chile; E-Mail: elquispe@unap.cl; 3 Laboratorio de Cultivo Celular, Facultad de Ciencias de la Salud, Universidad de Talca, Casilla 747, Talca 3460000, Chile; E-Mail: ctheodul@utalca.cl; 4 Laboratorio de Investigación de Productos Naturales (LIPRON), Instituto de Química del NOA (INIQUINOA.CONICET), Universidad Nacional de Tucumán, San Miguel de Tucumán 4000, Argentina; E-Mails: jorgelinaperezbasa@gmail.com (M.J.P.); asolecue@yahoo.com (A.S.C.); misla@tucbbs.com.ar (M.I.I.)

**Keywords:** *Prosopis chilensis*, mesocarp flour, flavonoids, antioxidant, traditional food

## Abstract

In South America, the mesocarp flour of *Prosopis* species plays a prominent role as a food resource in arid areas. The aim of this work was the characterization of the phenolic antioxidants occurring in the pod mesocarp flour of Chilean *Prosopis*. Samples were collected in the Copiapo, Huasco and Elqui valleys from the north of Chile. The samples of *P. chilensis* flour exhibited a total phenolic content ranging between 0.82–2.57 g gallic acid equivalents/100 g fresh flour weight. The highest antioxidant activity, measured by the DPPH assay, was observed for samples from the Huasco valley. HPLC-MS/MS analysis allowed the tentative identification of eight anthocyanins and 13 phenolic compounds including flavonol glycosides, *C*-glycosyl flavones and ellagic acid derivatives. The antioxidant activity and the phenolic composition in the flour suggest that this ancient South American resource may have potential as a functional food.

## 1. Introduction

Worldwide climate change and the long-lasting drought in northern Chile suggest that attention should be paid to native species that are adapted to arid environments. In arid and semi-arid lands, pods of the trees belonging to genus *Prosopis*, locally known as “algarrobo” in South America, are relevant food sources [1]. They were gathered by all the pre-Columbian human groups, including those living in the south of United States of America [2], Amerindians in the Paraguayan Chaco [3,4], Argentina [5], and Chile [6,7]. *Prosopis* pods constitute a food source for humans and animals [8]. Different food products are prepared from *Prosopis*, including flour, sweets, syrup or fermented alcoholic beverages [6,8]. After the European conquest and introduction of new crops, “algarrobo” pods were used to feed cattle and sometimes used in the local cuisine. The traditional use remained in rural areas and in the Chaco phytogeographical region of South America [3,4]. The pod flour is used to prepare a kind of bread, known as “patay” in Argentina [9] or is fermented into various alcoholic beverages (“añapa”, “aloja” and “chicha”) [10].

In northern Chile and Peru, “algarrobo” pods were an important food source in pre-Hispanic times and they can be found in archeological sites and in burials [11]. In Chile, the commercialized “algarrobo” flour is mainly imported from Peru by small local businessmen. The total production of “algarrobo” flour in Peru was estimated in 1422 tons in 2011 [12]. According to Soto and Gysling [13] the cultivated area of “algarrobo” trees in Peru comprises 5140 ha, mostly located in the Tarapaca Region (3246 ha), close to the border with Chile. Data about “algarrobo” flour production in Chile is not available. The *Prosopis* pods mesocarp flour was investigated for its food applications [14] and has been used to prepare cookies and fried chips [10]. The *Prosopis* flour potential as food was revised by Felker *et al*. [15] and by Cardozo *et al*. [9].

Chilean and Argentinean *Prosopis* species were investigated for their alkaloid content and composition, as well as for some biological activities, including enzyme inhibition, antioxidant effect and DNA binding [9,16,17,18]. The alkaloids isolated so far occur mainly in the leaves (folioles) while the pods contained large amounts of the amino acid proline, as should be expected for plants growing in soils affected by drought and salinity [19,20,21]. *Prosopis* pods were also analysed in a study on the proximate composition and bioactivity of food plants consumed by Chilean Amerindians [7]. Despite its long tradition of use as food and the potential of local resources for cuisine, there is little information on the phenolic compounds that can occur in the Chilean “algarrobo” pods mesocarp flour. The Argentinean “algarrobo” pods meal [5,9] as well as the seed flour [22] was investigated. The phenolic from “algarrobo” pods syrup was described by Quispe *et al.* [23]. Phenolics occurring in plant foods, grains and flour are dietary constituents that have been shown to present relevant biological activities. The pod flour of the Argentinian *Prosopis alba* and *P. nigra* contains antioxidant and anti-inflammatory agents [5], are not genotoxic and can be considered safe for human consumption [9]. It has been shown that the wheat flour contains antioxidant and antiproliferative phenolics [24] while beans phenolics shows antioxidant effects and are inhibitors of the enzymes α-amylase and α-glucosidase [25]. Lentil phenolics are not only antioxidant, but also inhibit α-glucosidase and pancreatic lipase [26]. Methanolic extracts from raw and processed Kenyan native food ingredients disclosed the antioxidant and hypoglycemic potential of their phenolic constituents [27]. In continuation of our studies on native South American food resources, we now report the naturally occurring phenolic compounds and antioxidant activity of Chilean *Prosopis* pods mesocarp flour.

## 2. Results and Discussion

### 2.1. Prosopis Flour Characterization and Antioxidant Activity

**Figure 1:**
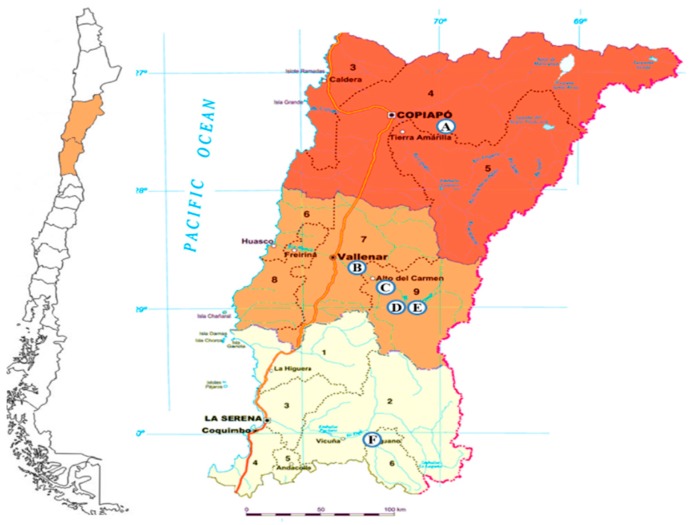
Map of Chile showing the collection places of *Prosopis* pods. Copiapo valley: Puquio (A); Huasco valley: Alto del Carmen (B), El Transito (C); Pinte (D) and Plaza de Pinte (E); Elqui valley (F).

**Figure 2:**
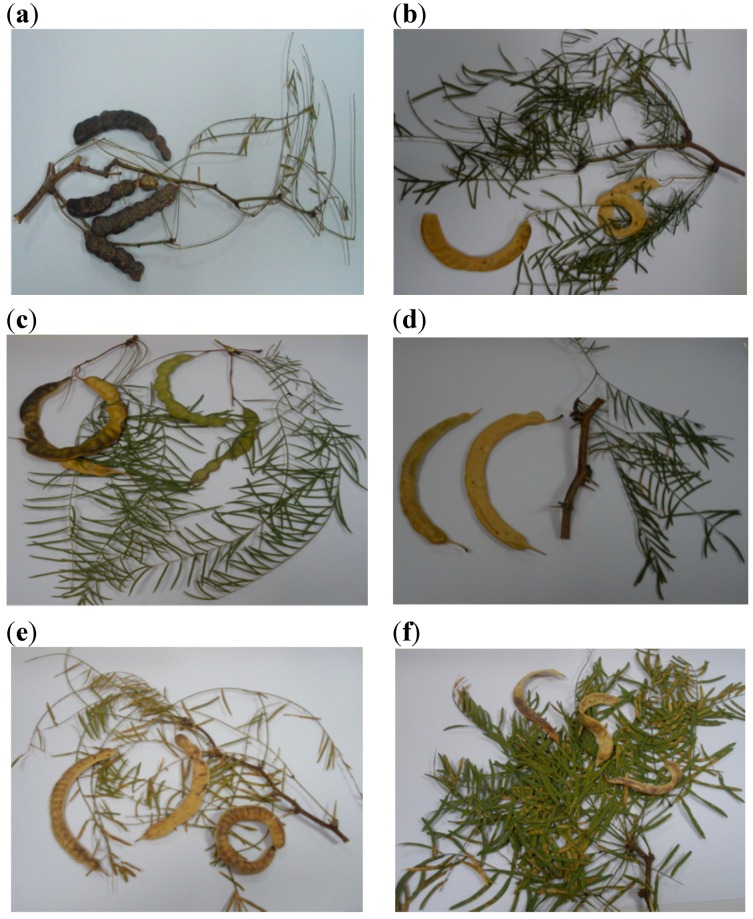
Chilean *Prosopis* pods showing morphological variation according to collection place. Puquio (**a**); Alto del Carmen (**b**), El Transito (**c**); Pinte (**d**); Plaza de Pinte (**e**); Elqui valley (**f**).

Six “algarrobo” pods samples were collected from the longitudinal valleys of Huasco, Elqui and Copiapó in northern Chile (Figure 1). The morphological variation of the samples is shown in Figure 2. The flour yield, phenolic and flavonoid content and antioxidant activity of the flour extracts were determined and are summarized in Table 1. The *Prosopis* flours presented different hues according to the colour of the pods. Pods from Elqui valley showed sticky granules producing tacky flours when ground. The percent of mesocarp flour yield ranged from 36.7% flour to pod ratio for the deep purple Puquio sample, 31.8% for the Elqui valley and 3.3%–17.8% for the beige pods of *P. chilensis* collected in the Huasco valley, respectively. The sample from El Transito, Huasco Valley, presented a very low flour to pod ratio (3.3%). With thin pods, this sample is considered inedible by the local population [3,4,8]. 

**Table 1:** Percent flour yield from pods, total phenolic (TP) and flavonoid (TF) content in flour weight (FW) and antioxidant activity of phenolic enriched methanol flour extract (PEFE) of Chilean *Prosopis* mesocarp flour.

Sample Origin	% Flour to Pod Ratio	Flour Color	TP(g GAE/100 g FW)	TF(g QE/100 g FW)	% XAD-Retained PEFE	DPPH SC_50_ (µg PEFE/mL)	FRAP (mMoles TE/g PEFE)	TEAC (μM TE/g PEFE)
*Copiapó valley*								
Puquio	36.7	Light greyish tan	2.54 ± 0.12	n.d. ^a^	4.26	70.51	0.50 ± 0.01	267.5
*Huasco valley*								
Alto del Carmen	15.4	Light tan	2.57 ± 0.09	0.38 ± 0.07	0.19	12.07	3.45 ± 0.06	3206.6
El Tránsito	3.3	Light tan	1.33 ± 0.06	0.25 ± 0.01	1.63	52.85	0.65 ± 0.04	428.6
Pinte	15.5	Light tan	0.82 ± 0.03	0.17 ± 0.01	0.91	52.97	0.63 ± 0.06	530.5
Plaza de Pinte	17.8	Light tan	2.11 ± 0.10	0.56 ± 0.10	0.10	23.74	1.21 ± 0.08	Inactive
*Elqui valley*								
Elqui valley	31.8	Pale tan	0.89 ± 0.11	0.23 ± 0.03	2.08	>100	0.36 ± 0.01	Inactive
Quercetin ^b^						7.82 ± 0.30	10.77 ± 0.16	8157.9

Abbreviations. FW: Fresh flour weight; PEFE: phenolic.enriched MeOH flour extract; TP: total phenolic content; TF: total flavonoid content; Antioxidant activity: DPPH (discoloration of the free radical 1,1-diphenyl-2-picrylhydrazyl, SC_50_ in µg PEFE/mL), FRAP (Ferric Reducing Antioxidant Power, mMol TE/g PEFE), TEAC (Trolox-Equivalent Antioxidant Capacity, μM TE/g PEFE) were carried out in triplicate and results are expressed as mean values ± SD. ^a^ below quantification level. ^b^ Quercetin at 30 µg/mL for FRAP assay.

The range of phenolic compounds (TP) in flours was 0.82–2.57 g GAE per 100 g FFW. The higher values were from Alto del Carmen (2.75 g GAE/100 g FFW), Puquio (2.54 g GAE/100 g FFW) and Plaza de Pinte (2.11 g GAE/100 g FFW). Total flavonoid (TF) content in flour was low (0.17–0.56 g QE/100 g flour weight), and no correlation was observed between the TP and TF content. As TP and TF of the flour was low, samples were enriched in phenolics for antioxidant activity studies and phenolic profiling. The flour samples were extracted with MeOH and phenolics were retained on Amberlite XAD-7 to obtain the phenolic-enriched flour extract (PEFE). The highest PEFE was from the Copiapo Valley sample (4.26%). Lower PEFE values for the different samples of *P. chilensis* ranged from 0.10% to 2.08% for the Huasco and Elqui Valley samples (Table 1).

The best antioxidant activity of the PEFE, measured by the DPPH discoloration assay, was found in the Huasco Valley samples: Alto del Carmen and Plaza de Pinte (SC_50_ 12.07 and 23.74 µg/mL, respectively). The same samples presented the highest activity in the FRAP assay, with values of 3.45 and 1.21 mM TE/g PEFE, respectively. In addition, the highest TEAC value was observed for the Alto del Carmen sample with a 3206.61 µM TE/g PEFE. There was a statistical correlation between the TP content and FRAP (*r* = 0.565; *p* < 0.05). Large variation in mesocarp flour yield and phenolic content was observed for *P. chilensis*. The close related species *P. alba* and *P. nigra* are common in the Chaco zone of South America and occur in the eastern Andean ranges of Argentina.

In a study by Cardozo *et al.* [9], TP in ethanolic extracts of *P. alba* and *P. nigra* pods flour were 0.18 and 0.19 g/100 g dry weight (DW), with flavonoids accounting for 0.01 and 0.06 g/100 g DW, respectively. When the flour was extracted with water, the TP values increased to 0.40 and 0.41 g/100 g DW and TF to 0.03 and 0.13 g/100 g DW for *P. alba* and *P. nigra*, respectively. The TP (0.82–2.57 g GAE/100 g flour) and TF content (0.17–0.56 g QE/100 flour) of the Chilean *Prosopis* flour samples was higher than that of the Argentinian species. 

In a review on pod mesocarp flour of *Prosopis* species, Felker *et al.* [14] refer to free phenolic concentrations of 0.18 and 0.41 GAE/100 g DW for *P. alba* and *P. nigra* flour, respectively. According to the same author, TP for wheat bran ranges between 0.126–0.316 g GAE/100g DW or 0.27–0.35 g GAE/100 g DW while white wheat flour contains 0.0044–0.014 g GAE/100g DW [14]. 

### 2.2. HPLC-DAD-MS/MS Analysis

**Figure 3:**
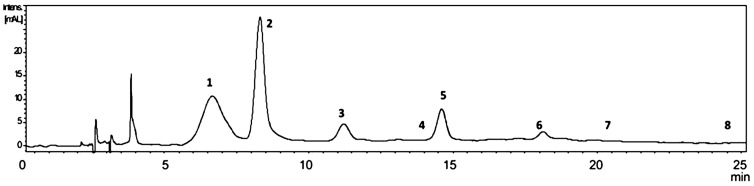
HPLC-DAD chromatogram of the phenolic enriched flour extract (PEFE) of Puquio showing the anthocyanins occurring in the sample. Detection: 535 nm. Compounds: **1**: Cyanidin 3-hexoside; **2**: Cyanidin 3-hexoside; **3**: Peonidin 3-hexoside; **4**: Petunidin hexoside; **5**: Cyanidin malonyl hexoside; **6**: Peonidin malonyl hexoside; **7**: Peonidin 3-hexoside; **8**: Malvidin hexoside.

**Figure 4:**
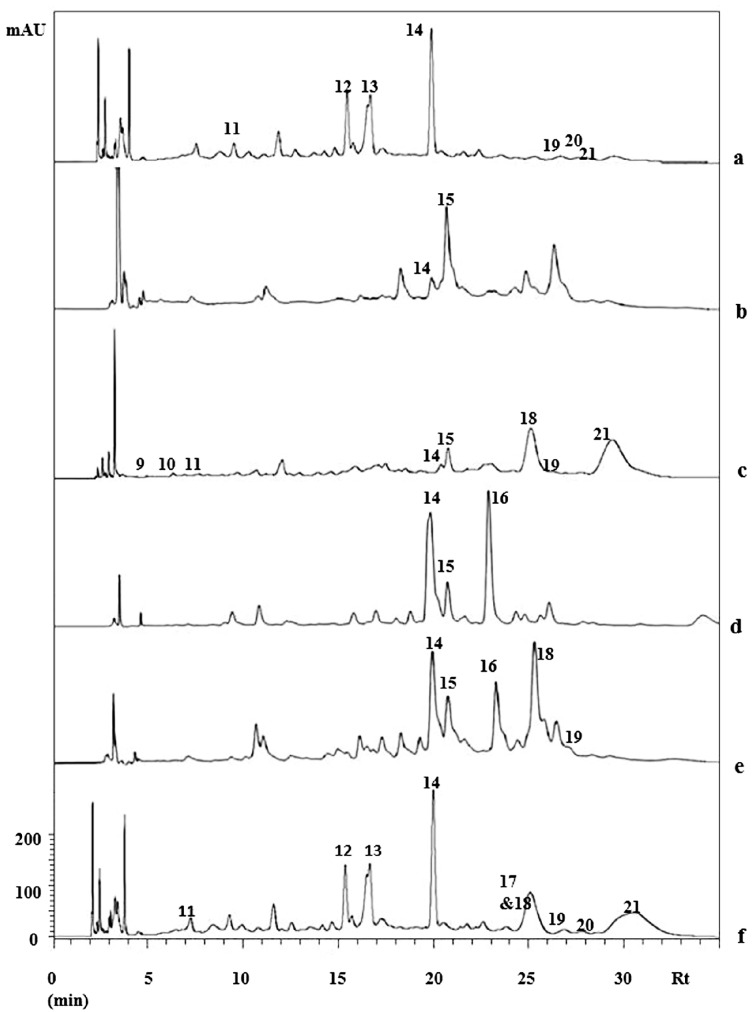
HPLC-DAD chromatogram of the Amberlite-retained fraction from the methanolic extract from “algarrobo” mesocarp flour. a: Puquio; b: Alto del Carmen; c: El Transito; d: Pinte; e: Plaza de Pinte; f: Elqui valley. Detection: UV, 254 nm. Compounds: **9**: Ellagic acid (EA) hexoside; **10**: Hydroxyferuloyl hexoside; **11**: EA hexoside; **12**: Vicenin II/Isomer; **13**: Vicenin II/Isomer; **14**: Schaftoside/isoschaftoside; **15**: Quercetin (Q) dihexoside; **16**: Q-hexosidepentoside; **17**: Q-methyl ether rhamnoside hexoside; **18**: Q-rutinoside; **19**: Isovitexin; **20**: Q-rhamnoside-hexoside; **21**: Q-methyl ether rhamnoside hexoside.

**Figure 5:**
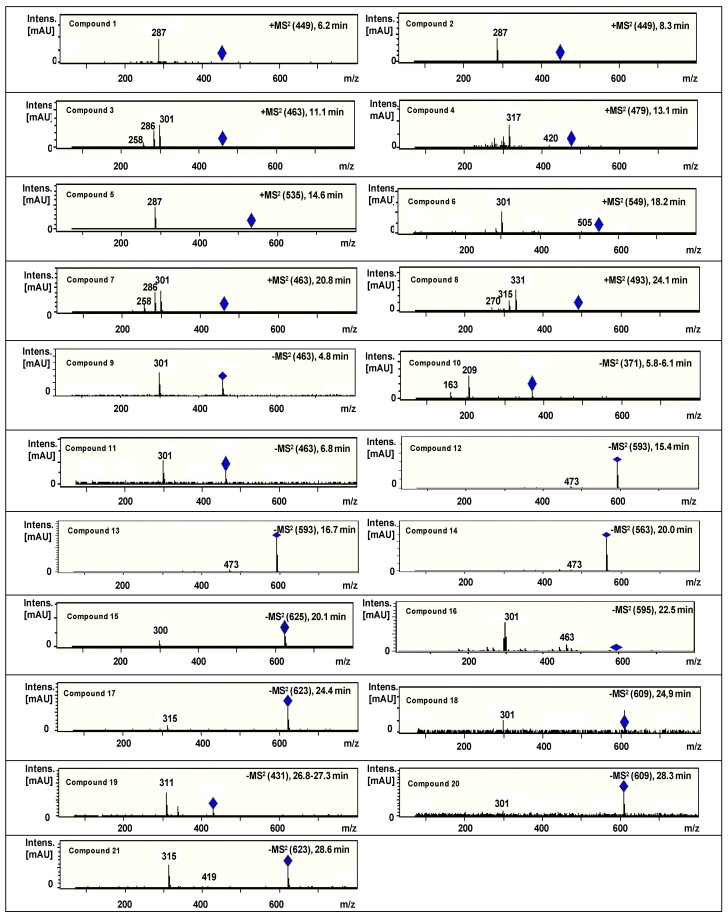
Extracted ion MS^2^ spectra of compounds **1–21** tentatively identified in the phenolic-enriched flour extract of Chilean *Prosopis* pods.

The composition of PEFE was assessed by HPLC-DAD-MS/MS^n^. Anthocyanins were detected in the positive ion mode, while other phenolic compounds were analyzed in the negative ion mode. The representative HPLC-DAD chromatogram of PEFE from the Puquio sample is presented in Figure 3. The HPLC-DAD chromatograms of other samples are shown in Figure 4. Extracted ion MS^2^ spectra of compounds **1–21** are presented in Figure 5. The tentative identification of anthocyanins (A) and phenolic compounds (B) is presented in Table 2. 

The visible spectra of compounds **1–3**, **5–7**, as detected in the DAD, show maxima in the 516–520 nm range, in agreement with anthocyanidins bearing a substituent at the 3-position [28,29]. According to Ververidis *et al.* [30] 3-glycosylation of the anthocyanidins confers stability to the pigments, being the most common substitution pattern for this group of compounds. MS/MS experiments show loss of 162 atomic mass units (amu) for compounds **1–8**, leading to a *m/z* ion at 287 amu for compounds **1**, **2** and **5**, *m/z* 301 for **3**, **6** and **7**, *m/z* 317 for **4** and 331 for **8**. This is consistent with the occurrence of cyanidin, peonidin, petunidin and malvidin hexosides in the sample. The main compound was cyanidin 3-hexoside. A malonoyl unit was detected in compounds **5** and **6**, assigned as cyanidin malonyl hexoside and peonidin malonyl hexoside, respectively. 

These anthocyanins have not been previously reported from *Prosopis chilensis* and are responsible, at least in part, of the dark purple colour of the pods. Compounds **9** and **11** presented the same pseudo molecular ion at 463 amu and loss of a hexose, leading to the ion at *m/z* 301. The elution time and UV spectrum of **11** is in agreement with a phenolic compound, consistent with ellagic acid. Both compounds differ in the identity of the hexose and were tentatively identified as ellagic acid hexosides. The compound **10** showed in the MS/MS spectrum the loss of a hexoside, leading to the aglycone at *m/z* 209, in agreement with hydroxyferulic acid hexoside [31].

The UV spectra of compounds **15–18**, **20** and **21** show maxima in the range 349–354 nm, in agreement with the Band I of flavonol substituted at the 3-*O*-position. MS/MS analysis showed the loss of two 162 amu fragments (hexose) for compounds **15** and one hexose and a pentose for **16**. 

Consecutive losses of rhamnose and hexose or rutinoside were observed for **17**, **18**, **20** and **21**, leading to aglycones with *m/z* 301 or 315, in agreement with quercetin (**15**, **16**, **18** and **20**) and quercetin methyl ether (**17** and **21**). The compounds were identified as quercetin glycosides **15**, **16**, **18** and **20** and quercetin methyl ether glycosides **17** and **21**. Flavone *C*-glycosides show characteristic UV maxima around 330 nm (Band 1) and losses of 120 amu in the MS/MS spectra [32]. Compounds **12–14** and **19** presented UV and mass spectra in agreement with this group of compounds and were assigned as vicenin II isomers (**12** and **13**), schaftoside or isoschaftoside (**14**) and isovitexin (**19**). The *C*-glycosyl flavones schaftoside, isoschaftoside and vicenin II were recently identified as constituents of *Prosopis* pods syrup [23].

Table 2Tentative identification of anthocyanins and phenolics in phenolic-enriched *Prosopis* pod mesocarp flour extracts by HPLC-DAD-ESI-MS data. Anthocyanins (**A**) were detected in Puquio sample. Phenolics different than anthocyanins (**B**) were found in all phenolic-enriched pod mesocarp flour extracts.

**Table:** **A**: Anthocyanins in *Prosopis* pods meal (Puquio sample), detection at 535 nm.

Compound	R_t_ (min)	UV λ_max_ (nm)	[M]^+^ (*m*/*z*)	MS/MS (*m*/*z*)	Tentative Identification
**1**	6.2	516, 425 sh, 279, 224	449	287	Cyanidin 3-hexoside
**2**	8.3	516, 425 sh, 279, 224	449	287	Cyanidin 3-hexoside
**3**	11.1	517, 321 sh, 278, 225	463	301,286,258	Peonidin 3-hexoside
**4**	13.1	-	479	420,317	Petunidin hexoside
**5**	14.6	517, 307sh, 285, 228	535	287	Cyanidin malonyl hexoside
**6**	18.2	517, 320 sh, 279, 228	549	505,301	Peonidin malonyl hexoside
**7**	20.8	520, 319 sh, 270, 229	463	301,286,258	Peonidin 3-hexoside
**8**	24.1	-	493	331,315,270	Malvidin hexoside

**Table:** **B**: Phenolics in phenolic-enriched *Prosopis* pod mesocarp flour extracts, detection at 254 nm.

Compound	R_t_ (min)	UV λ_max_ (nm)	MW	[M-H] ‾ and Fragment Ions	Tentative Identification
**9**	4.8	-	464	463, 301	Ellagic acid hexoside
**10**	5.8–6.1	289, 228	372	371, 209, 163	Hydroxyferulic acid hexoside
**11**	6.8	278	464	463, 301	Ellagic acid hexoside
**12**	15.4	334, 270	594	593, 473	Vicenin II/Isomer
**13**	16.7	334, 271	594	593, 473	Vicenin II/Isomer
**14**	20.0	333, 270	564	563, 503, 473, 443, 383	Schaftoside/isoschaftoside
**15**	20.1	352, 262	626	625, 300	Q-dihexoside
**16**	22.5	352, 267 sh, 256	596	595, 463, 301	Q-hexosidepentoside
**17**	24.4	354, 268 sh, 254	624	623, 315	Q-methyl ether rhamnoside hexoside
**18**	24.9	355, 297sh, 267sh, 254	610	609, 301	Q-rutinoside (rhamnoside hexoside)
**19**	26.8–27.3	-	432	431, 311	Isovitexin
**20**	28.3	352, 265 sh, 254	610	609, 301	Q-rhamnoside-hexoside
**21**	28.6	349, 267 sh, 253	624	623, 419, 315	Q-methyl ether rhamnoside hexoside

HPLC-DAD-MS/MS analysis of the PEFE allowed the tentative identification of eight anthocyanins, two ellagic acid glycosides, six flavonol *O*-glycosides, four flavone *C*-glycosides and a hydroxyferulic acid hexoside. Anthocyanins were detected only in the sample from the Copiapo Valley (Puquio). The compounds were cyanidin, peonidin, petunidin and malvidin derivatives, suggesting similarity with *P. nigra* [5]. Anthocyanins have been identified in several crops, including black beans [33], black rice [34], and purple corn [35]. The anthocyanins delphinidin 3-glucoside, petunidin 3-glucoside and malvidin 3-glucoside were reported for black bean [33] and cyanidin-3-*O*-sambubioside, the 3-*O*-glucosides of delphinidin, cyanidin, pelargonidin and peonidin were described from the immature purple pods of yard-long beans [36]. Despite their widespread distribution in Nature, anthocyanins have not been reported from South American *Prosopis* pods until a recent study on *P. alba* and *P. nigra* mesocarp flour [5]. In the present work, anthocyanins were identified in the *Prosopis* sample collected in Puquio. This place was a natural corridor that allowed crossing the Andes from the Copiapo Valley to the Argentinian provinces of Catamarca, La Rioja and Tucuman through the Paso San Francisco. As this was also a pre-Columbian route to cross the Andes and there is no previous records of *P. nigra* in Chile, we cannot rule out three possibilities: (i) that the distribution range of the species included part of the western Andean valleys; (ii) that the species was introduced in Chile by migrants or, (iii) that the sample is a high anthocyanin variety of *P. chilensis*. The pods as well as the starch from *P. chilensis* and *P. flexuosa* are frequently found in archeological remains in the dry valleys of Catamarca, Argentina [11]. 

Main constituents in the PEFE were flavonoids. Different patterns of phenolic compounds other than anthocyanins were observed in the chromatograms of the samples (Figure 4). Similar constituents are present in the Puquio and Elqui samples with the *C*-glycosylflavones **12**, **13** and **14** as the main compounds. In the Pinte, Plaza de Pinte and Alto del Carmen flours (Figure 4), the *C*-glycoside **14** occurs with the quercetin glycoside **15**, but in different ratios. The Plaza de Pinte sample showed a more complex composition, with additional quercetin glycosides. The sample from El Transito presented a higher proportion of **18** and the flavonol glycoside **21**. Quercetin glycosides and flavone *C*-glycosides are observed in the PEFE. The chemistry and morphology of the *Prosopis* sample from the Copiapo valley suggest a species different than *P. chilensis*. 

The lactone 5,6-dihydro-6-propyl-2*H*-pyran-2-one was identified as the major volatile from *Prosopis* flour [37].The alkaloids phenethylamine and tryptamine were isolated from *P. chilensis* pods [6]. In the present work, only phenolics were identified in the PEFE. The phenolics from the pods mesocarp meal were compared with those identified in the traditional syrup “algarrobina” [23]. Common constituents were flavone *C*-glycosides such as vicenin II or isomer, schaftoside/ isoschaftoside and quercetin glycosides, suggesting a common trend in the phenolic compounds composition of *Prosopis* pods-derived products. The total flavonoid *C*-glycosides from *Abrus mollis* extract, containing mainly vicenin-II, isoschaftoside and schaftoside was shown to display strong anti-inflammatory and hepatoprotective effect in mice [38]. The *C*-glycoside mixture also presented antioxidant and protective effect on lipopolysaccharide-induced lipotoxicity in mice [39]. The same compounds occur in the phenolic-enriched fraction of the *Prosopis* flour. Vicenin-II shows anti-glycation activity [40] and is active against prostate cancer cells [41]. The structural features of flavonoids associated with antioxidant effect are known [42]. Tsuchiya [43] reported the structural relationship between flavonoids and cell-mimetic membranes and its relation with antioxidant and anti-proliferative effect on cells. However, increasing evidence indicates that the *in vivo* bioactive flavonoids are not necessarily the naturally occurring compounds [44]. The *O*-flavonoid glycosides are hydrolyzed and the aglycones are transformed into glucuronides, *O*-methylated derivatives and sulphates and are further degraded by the gut microflora [44].

The phenolics identified in the flour are compounds with known antioxidant properties and display also other biological effects that might have a positive impact in the health of consumers. More research work should be undertaken to fully disclose the potential of this ancient South American resource as functional food.

## 3. Experimental Section 

### 3.1. Chemicals

Folin-Ciocalteau phenol reagent, 2,4,6-tri(2-pyridyl)1,3,5-triazine (TPTZ) sodium acetate, 1,1-diphenyl-2-picrylhydrazyl radical (DPPH), quercetin, gallic acid and AlCl_3_ were purchased from Sigma-Aldrich (St. Louis, MO, USA). 2,2’-Azino-bis(3-ethylbenzothiazoline-6-sulfonic acid (ABTS) diammonium salt, 6-hydroxy-2,5,7,8-tetramethylchroman-2-carboxylic acid (Trolox), potassium persulfate, sodium carbonate, FeCl_3_·6H_2_O, HPLC-grade methanol, acetonitrile and formic acid were purchased from Merck (Darmstadt, Germany). Ultrapure water was obtained using a BarnstedEasyPure water filter (Thermo Scientific, Marietta, OH, USA).

### 3.2. Plant Material and Sample Preparation

Algarrobo pods were collected in the Copiapo, Huasco and Elqui valleys in February 2013 (Figure 1). The samples were classified as *Prosopis chilensis* by Dr. Patricio Peñailillo and voucher specimen were deposited at the Herbario de la Universidad de Talca. The collection places were as follows: Copiapo Valley: road to Paso San Francisco, near Puquio (27°08′55′′S, 69°52′24′′W); Huasco Valley: Alto del Carmen (28°44′50′′S, 70°29′57′′W), El Transito (28°51′04′′S, 70°18′33′′W); road to Pinte (28°57′47′′S, 70°16′54′′W) and Plaza de Pinte (28°58′45′′S, 70°16′55′′W); Elqui Valley: 30°06′39′′S, 70°29′58′′W. Samples were transported to the lab and kept at room temperature, according to the traditional storage indications. The air-dried pods were processed in a grinder to separate the seeds from the mesocarp flour. The traditional flour preparation was followed, using a mortar and pestle. The flour was sieved and weighed to establish the pod/mesocarp flour ratio. Pods flour was extracted with MeOH under sonication (2 × 3 min each time), in 1:10 flour to MeOH *w*/*v* ratio. The MeOH solution was filtered and taken to dryness under reduced pressure to afford the crude MeOH extract. The extracts were dissolved in water, filtered and adsorbed into Amberlite XAD-7, pre-treated as described in Jiménez-Aspee *et al.* [45]. Phenolic compounds were desorbed from the resin using MeOH and MeOH:H_2_O 7:3 (*v*/*v*) and the combined extracts of each sample were taken to dryness and lyophilized. The phenolic-enriched flour extracts (PEFE) were concentrated under reduced pressure and lyophilized for its analysis.

### 3.3. Total Phenolic (TP) and Total Flavonoid (TF) Contents 

The total phenolic (TP) and total flavonoid (TF) content was determined in the flour MeOH extract as described by Jiménez-Aspee *et al.* [45], with slight modifications. Stock solutions (2 mg/mL) were prepared in MeOH:H_2_O (1:1). For TP, the Folin-Ciocalteu method was followed. The results are expressed as g gallic acid equivalents (GAE)/100 g fresh flour weight (FFW). For TF, the AlCl_3_ methodology was used. TF was expressed as g quercetin equivalents (QE)/100 g FFW. Absorbance of each solution were measured by spectrophotometer (Thermo Spectronic Helios Alfa, Cambridge, UK) at 725 and 510 nm, respectively, after 15 min of incubation at room temperature.

### 3.4. Antioxidant Activity

The antioxidant activity of the samples was determined by three assays, as described [45,46]. PEFEs were dissolved in 50% *v*/*v* aqueous methanol at a final concentration of 300 μg/mL. Stock solutions were filtered and kept in the dark, and all analyses were performed on the same day.

DPPH discoloration assay was carried out with final concentrations of 100, 33 and 11 μg/mL. The DPPH solution was freshly prepared in methanol (20 mg/L) and mixed with the extract at the above given concentrations. Absorbance was measured at 517 nm in a universal microplate reader (Biotek Instruments Inc., ELx 800, Winooski, VT, USA). SC_50_ values (μg/mL), corresponding to the amount of extract that scavenges the radical concentration by 50%, were calculated using the OriginPro 8.0 software (OriginLab Corporation, Northampton, MA, USA). 

For the ferric reducing antioxidant power (FRAP) assay, a 300 μg/mL extract aliquot was mixed with warm FRAP solution and left to stand in the dark for 30 min. Absorbance was read at 593 nm using a spectrophotometer (Thermo Spectronic Helios Alfa) Results are expressed as mMoles Trolox equivalents (TE)/g extract.

The Trolox-equivalent antioxidant capacity (TEAC) determinations were carried out by mixing ABTS^•+^ with fresh standard (1 mM Trolox) or extract (100, 150, 200, 250 and 300 µg/mL). Absorbances were read at 734 nm after 6 min of room temperature incubation using a spectrophotometer (Thermo Spectronic Helios Alfa). Results are expressed as μM Trolox equivalents/g extract.

### 3.5. HPLC-DAD-MS Analysis

The extracts were analysed by HPLC coupled to a diode array detector (HPLC-DAD) to set the conditions for HPLC-ESI-MS/MS studies. The HPLC system used for DAD analysis was a Shimadzu equipment (Shimadzu Corporation, Kyoto, Japan) consisting of a LC-20AT pump, a SPD-M20A UV diode array detector, CTO-20AC column oven and a LabSolution software. A MultoHigh 100 RP 18–5µm (250 × 4.6 mm) column (CS-Chromatographie Service GmbH, Langerwehe, Germany) maintained at 25 °C was used. Approximately 5 mg/mL of PEFE was filtered through a 0.45 µm filter (Waters, Milford, MA, USA) and injected into HPLC-DAD and HPLC-ESI-MS/MS. The compounds were monitored at 254, 330 and 535 nm, and UV spectra from 200 to 600 nm were recorded for peak characterization. The HPLC analysis was performed using a linear gradient solvent system as described by Quispe *et al.* [23]. The flow rate was 1 mL/min and the volume of injected sample was 20 µL.

The mass spectrometer consisted of a HPLC HP1100 (Agilent Technologies Inc., Santa Clara, CA, USA) connected through a split to the mass spectrometer Esquire 4000 Ion Trap LC/MS(n) system (Bruker Daltonik GmbH, Bremen, Germany). Ionization was performed at 3000 V assisted by nitrogen as nebulizing gas at 24 psi and as drying gas at 365 °C and a flow rate of 6 L/min. Negative ions were detected using full scan (*m/z* 20–2200) and normal resolution (scan speed 10300 *m*/*z*/*s*; peak with 0.6 FWHM/*m*/*z*). The trap parameters were set in ion charge control (ICC) using manufacturer default parameters, and maximum accumulation time of 200 ms. Collision induced dissociation (CID) was performed by collisions with helium background gas present in the trap and automatically controlled through Smart Frag option.

Additional mass spectrometry measurements were performed using an Agilent Series 1200 LC System (Agilent, Ramsey, MN, USA) coupled to a MicroQTOF Q II (Bruker Daltonics, Billerica, MA, USA). The HPLC system consisted in a micro vacuum degasser, binary pumps, an autosampler (40 μL sample loop), a thermostated column compartment and a diode array detector. The mass spectrometer equipped with an electrospray ion source and QTOF analyser, was used in MS and MS/MS mode for the structural analysis of phenolics. HPLC analyses were performed on a thermostated (40 °C) MultoHigh 100 RP 18–5µm (250 × 4.6 mm) column (5 μm) with a flow rate of 1.0 mL/min using a split to the detector. The solvents and ramp were the same as described for the ion trap equipment. 

ESI-MS detection was performed in negative and positive ion mode with mass acquisition between 100 and 1500 Da. Nitrogen was used as drying and nebulizer gas (7 L/min and 3.5 bar, respectively), and 180 °C for drying temperature. For MS/MS experiments, fragmentation was achieved by using Auto MS^2^ option. DAD analyses were carried out in the range between 200 and 700 nm. The identification of phenolic compounds in “algarrobo” pods meal was carried out by comparison of the spectral properties (UV and ESI-MS and MS/MS) of the compounds with literature data. 

### 3.6. Statistical Analysis

Determinations of TP, TF, DPPH and FRAP, were performed in triplicate and results are expressed as mean values ± SD. For the TEAC assay, a curve was plotted for each sample and a correlation coefficient with 95% confidence limit was established. To assess the relationship between the antioxidant activities and the TP and TF content, Pearson’s correlation coefficients were calculated with 95% confidence. Statistical analysis was carried out using the software SPSS 14.0 for Windows.

## 4. Conclusions 

The main compounds in the PEFE were flavonoids. One sample contained cyanidin hexoside and other anthocyanins, being this the first report on the occurrence of anthocyanins in Chilean *Prosopis* pods. The phenolic composition and antioxidant properties of the Chilean *Prosopis* mesocarp flour supports its use as a functional food. Additional studies are required to compare the potential of the different flour sources in artisanal and commercial food products. A higher number of samples should be analyzed to have a better picture on the phenolic composition of Chilean *Prosopis* mesocarp flour. 
